# The Hospital. Nursing Section

**Published:** 1902-09-13

**Authors:** 


### The Hospital.

### Nursing Section.

Contributions for this Section of "The Hospital" should be addressed to the Editor, "The Hospital" Nursing Section, 28 & 29 Southampton Street, Strand, London, W.C.

No. 833.—Vol. XXXII. *SATURDAY, SEPTEMBER* 13, 1902.

### Notes on News from the Nursing World.

### THE KING'S NURSES.

Prior to his departure for Scotland, the King, we understand, sent for Miss Haines and Miss Fletcher, the two nurses who attended him through his illness, and having personally thanked them, gave them each a handsome present. The Queen and the Princesses also presented the nurses with valuable gifts. Miss Fletcher, who is now enjoying a holiday among her friends at Ashton in Lancashire, received quite an ovation on her arrival from Wigan at Bryn station. In addition to a number of grown-up people, there was a large number of children from the schools in the district, who lined each side of the road and followed her at a distance to her father's house in Wigan Road, where they raised three hearty cheers for "the King's nurse."

### NURSES FROM SOUTH AFRICA.

The arrival of the following nurses from South Africa has taken place during the week:—On the *Syria*, Sisters G. M. Beesby and H. Fisher; on the *Buluwayo*, Sisters S. A. Fisher and F. G. Rhodes; on the *Dunvegan Castle*, Sisters M. J. McNair and A. S. Liell; on the *Plassy*, Sisters J. Armitstead and A. D. Barnett; on the *Bavarian*, Sisters A. C. Anderson, C. L. Travis, and A. B. McDonald. It is now definitely announced that none of the members of Queen Alexandra's Imperial Military Nursing Service will be required to return to South Africa.

### THE NURSING ARRANGEMENTS AT LAMBETH WORKHOUSE AND INFIRMARY.

At an inquest last Friday into the circumstances attending the death of William Parsons, an inmate of Lambeth Workhouse, who committed suicide while he was in the lavatory, the jury returned a verdict of "suicide during temporary insanity," and added a rider to the effect that more nurses should be employed in the workhouse, and that the wards were not properly supervised. The coroner, in the course of some subsequent remarks, though admitting that "he was not very conversant with the practice of night nursing," said he agreed with the jury that the wards were not properly supervised. But we think that both the jury and the coroner lose sight of the fact that the Lambeth Workhouse is not the Lambeth Infirmary, and that immediately any of the inmates of the former are found to be ill they are transferred from one building to the other. As the buildings in Renfrew Road and Brook Street adjoin one another, this is very easy, and there is no delay in making the transfer. There are two trained nurses and an attendant always on duty in the infirm wards in the workhouse, and at night a charge nurse, also trained. The latter pays periodical visits during the night to the wards of the able-bodied inmates, in order to see that they are properly cared for. The nursing in the infirmary is, of course, quite distinct from that in the workhouse, and as a proof of the adequacy of the staff under Miss Byles, the matron of the infirmary, we may say that the average of nurses to patients is above that of one to ten. The suicide of Parsons is a regrettable occurrence; but the Lambeth Guardians cannot reasonably be charged with not providing a sufficient number of nurses for the poor under their protection. In fact, so far as the able-bodied are concerned, we fail to see any excuse for a nurse being even asked to enter their dormitories.

### "DISTINGUISHED SERVICES."

From the United States we learn that the commanding officer of the First Reserve Hospital at Manila invited attention "to the distinguished services of Nurse Alice S. Kemmer in volunteering to undertake the care of two very serious cases of small-pox, she herself never having had the disease"; that the surgeon-general endorsed the suggestion of the commanding officer that there should be, "as a partial reward, official recognition of her services"; and that the general commanding the division of the Philippines recommended "that the especially meritorious services of this nurse to the army be fittingly recognised in general orders, as is the custom for gallant and commendable acts on the part of soldiers." We do not for a moment wish to under-rate the services of Miss Kemmer, of whom it is said that by her devotion the lives of the two patients were saved. But there is really nothing in the least remarkable in a nurse volunteering to attend small-pox patients. Hundreds of English nurses would be ready to volunteer if need arose, and assuming that they had been properly vaccinated, they would run very little risk of catching the disease.

### THE SUICIDE AT CRUMPSALL INFIRMARY.

The terrible case of the nurse at Crumpsall Workhouse Infirmary, respecting whose suicide details are given in another part of the paper, is a very pathetic affair. While there was no direct evidence forthcoming to show that Miss Asgodby was suffering from mental aberration, there was none to indicate any cause for worry or trouble. The character and capacity of the deceased were alike unquestionable, and no witness who was called had anything to say of her that was not complimentary. The verdict, in the circumstances, was the only one possible; but it leaves unsolved doubts as to the real cause of the sad act.

### A RULE THAT SHOULD BE ABOLISHED.

The majority of the Board of Guardians of Newport, Shropshire, have come to a very ungracious and unjust decision. Under the direction of the medical officer of the workhouse the master engaged a temporary nurse for the infirmary during the holiday of one of the permanent nurses; and at a subsequent meeting of the Board the chairman proposed that the action should be "disapproved." His reason for his proposal was that "it had always been the rule, when one of their workhouse officers was away for a holiday, that the remaining officers should divide the duties amongst them, and there had been no alteration of that rule." Miss Roddam, as a member of the Board, in vain protested that it was not right to expect a nurse to do the night and the day duties of the house. Without hearing the medical officer, the Guardians pronounced against the abolition of a rule which cannot be too strongly condemned in the interests of both the patients and the nurses at Newport Workhouse Infirmary.

### THE LUXURY OF THE MODERN HOSPITAL.

The Superintendent of the New York City Training School for Nurses congratulates herself that "the absence of the luxury of the modern hospital" is a feature of the institution of which she is head, and affirms that while there is sufficient to work with, "yet the nurse must exercise her ingenuity at times to supply wants in an emergency." Undoubtedly, as Miss Gilmour contends, this makes her valuable in private duty, "since no nurse has all the conveniences of a hospital." But the best nurse is, none the less, the one who can adapt herself to circumstances, and though prepared to make shift at a pinch, is capable of turning to account, in the interests of the patients, the most up-to-date appliances and conveniences.

### THE PASSING OF THE MALE NURSE.

Another interesting point in connection with the New York City Training School is that the authorities are gradually getting rid of male nurses. They are only, in this respect, following the example at Bellevue Hospital, where, as we announced some time ago, women are taking the place of men as head nurses in male wards. But the fact is an admission that the movement—which originated in 1886 when the male training school in connection with the City Hospital was started—has not been altogether the success which was anticipated.

### A NURSE'S STRUGGLE WITH A PATIENT.

Not less commendable than the conduct of the two nurses in Tyrone County Infirmary who recently prevented a patient from committing suicide is that of Miss Jane Cracknell, a nurse at University College Hospital, who did her best the other day to save the life of George Hopper, a plasterer. Hopper, a man of 43, was a patient in one of the upper wards, facing Gower Street, and refused on a certain morning to take his medicine from his nurse, Miss Cracknell. She left him for a couple of minutes when she heard a crash of glass, and then found that Hopper had broken a large pane of glass in the window above his head, and had his head and shoulders out of the hole. She at once seized his legs, and, with help, prevented him from falling into the street below. The patient had, however, so severely injured himself that he died within a few minutes, the heart trouble from which he was suffering having been accelerated by the strain and exertion involved in the act, which the house physician attributed to an attack of delirium.

### REACTIONARY PROPOSALS AT PLYMOUTH AND LYNN.

A fatuous recommendation on the part of the hospital committee of the Plymouth Board of Guardians has, happily, been set aside at a general meeting of the Board, though the majority in favour of the amendment was only two. The hospital committee, in the face of the recommendation of one of the medical officers that a nurse should be appointed to fill the vacancy caused by a resignation, decided that nothing should be done. Perhaps the reminder of Mr. Mill, that in case the Board refused to make the appointment the medical officer and superintendent nurse had power to ask for a temporary nurse, the employment of whom would be very costly, influenced some of his brother guardians to vote against the extraordinary recommendation of the hospital committee. At King's Lynn, a similar attempt has been made to reduce the infirmary staff; but there, we are glad to observe, the advocates of a reactionary policy were badly beaten, eleven voting for the amendment to the proposal that a vacancy on the nursing staff should not be filled, and three against it. At Lynn the Chairman of the Board of Guardians was on the side of progress, and said that he would strongly oppose reverting to the old style of pauper nurses. The medical officer had stated that they could not do with fewer than six nurses, and he supported the medical officer. As the rules stipulate that there should be one nurse to every ten patients, we are surprised that even three of the Lynn guardians should have been prepared to ignore the opinion of the medical officer.

### POPLAR AND STEPNEY SICK ASYLUM.

In the annual report of the clerk to the manager of the Poplar and Stepney Sick Asylum, just issued, we note a reference to the revision of the salaries of the staff nurses which, with the approval of the Local Government Board, the managers have increased. The report says that during the year the staff nurses were invariably recruited from the ex-probationer nurses who had obtained their certificate after the three years' training in the asylum, and continues, "Under the former regulations the managers having trained the nurses, other institutions reaped the benefit of their training by offering better inducements. Seventy-six candidates for the office of probationary nurse had applied for appointment during the year. Of these 23 were appointed; two left, not being strong enough for the work; 21 were otherwise unsuitable; 20 applications were withdrawn; and there were 10 remaining on the books at the close of the year, awaiting appointment." With regard to the examinations of probationers, 10 candidates passed in April, and six in October. It is interesting and satisfactory to learn that eight nurses passed the examination in June, 1901, in sick cookery in connection with the cookery classes held at the asylum under the auspices of the Technical Education Board of the London County Council, and 14 at the examination in March this year.

### NEW NURSES' HOME AT SHEFFIELD.

The Nurses' Home in connection with the Sheffield Workhouse Infirmary, at Fir Vale, is a handsome building. It is of red brick, with Stoke Hall stone dressings, and the roof is covered with Westmorland slates. There are separate bedrooms for about 50 nurses, a general tea-room and recreation-room, and special sitting-rooms for charge nurses. With the opening of the Home, and other extensive improvements, the Sheffield Infirmary will come into line with the other great provincial infirmaries.

### MAIDSTONE WORKHOUSE INFIRMARY.

A curious situation has arisen at Maidstone. There being no able-bodied inmates in the workhouse, that institution, like the infirmary attached to it, has become a home for sick persons, and one of the medical officers has expressed the opinion to the Local Government Board that the time has come when a superintendent nurse should be appointed, apart from the matron. As there are upwards of 100 patients in the infirmary we should, in ordinary circumstances, entirely agree with him. But, according to the Chairman of the Board, the matron herself is properly qualified to carry out the duties of superintendent nurse, and does, in fact, at present discharge them. If this be the case, and the matron has not too many demands upon her time to be able to act as superintendent, we do not see the necessity for the appointment suggested, though it may be needful to increase the nursing staff.

### A DISTRICT NURSE FOR NORTH ILLOGAN.

North Illogan, a large and important parish between the parishes of Camborne and Redruth, is, through the liberality and energy of Mrs. Bassel, of Tehidy, to be provided with a district nurse. The busy little port of Portreath, with numerous houses, is an important centre, also the villages of Bridge and Illogan Church town, while the inmates of the many scattered cottages and farmhouses will no doubt also gladly avail themselves in sickness of the service of a trained nurse. A penny per month will be considered a sufficient contribution from householders of the working classes, and a minimum of 5s. per annum from other subscribers with larger incomes. These contributions will, it is hoped, render the work self-supporting. The nurse will work under the direction of the County Nursing Association. Illogan is almost unique among Cornwall parishes in not having before engaged a district nurse, a fact which speaks for itself as showing how well residents of the county understand and appreciate trained nursing. At a time when help was much needed after a storm in which the steamship *Escuriel* became a complete wreck at Portreath, some few years ago, the hon. secretary of Redruth Nursing Association conveyed their nurse to this port and left her there in charge of severe cases several weeks—a civility which Illogan in due time recognised by a substantial payment. Now Illogan will have its own nurse in periods of wreck and difficulty.

### THE VALUE OF PENNY COLLECTIONS.

The importance of small contributions has always been insisted upon in these columns, and a striking practical illustration of how much may be done in this direction is shown by the amount raised for the Women's Memorial to Queen Victoria at the instance of a single lady. The Mayoress of Finsbury organised a penny collection, and thanks to the energy and persistence of the collectors a sum exceeding £100 has been obtained. It is true that to raise £100 24,000 calls may have been necessary, but divided among a number of persons the labour is not so great as it seems. In any case, the result cannot fail to be gratifying to those who have assisted in any way to bring it about.

### THE MIDWIFERY QUALIFICATION.

It will be noticed that the Richmond (Surrey) Board of Guardians, who require two nurses in place of one who leaves to get married and another who has obtained a better position, offer £30 to a nurse without midwifery qualification, and £35 to a nurse who holds a midwifery certificate. There is no doubt that the enactment of the Midwives' Registration Act will tend to enhance the marketable value of the possession of a midwifery certificate by a workhouse infirmary nurse.

### NURSES AND TITLES.

At the meeting of the New York State Nurses' Association to be held in Rochester next month the question of "properly graduated nurses" having some distinctive titles similar to those of doctors will be discussed. The following were suggested at a meeting in Utica :—G.N., graduate nurse; R.N., registered nurse; R.G.N., registered graduate nurse; and C.N., certified nurse. As only 13 members were present at the Utica gathering, it may be concluded that at the larger meeting still further suggestions will be forthcoming.

### PAYING PATIENTS AT LICHFIELD.

The committee of the Lichfield Nursing Home have decided to provide accommodation at the Home for paying patients, and the local press compliment them upon having conceived a very happy idea. The Home is not in connection with the Queen's Jubilee Institute for Nurses, and the Committee cannot therefore be accused of departing from the sound rule which the affiliated branches are required to observe. If the nurses attached to it were not engaged for the purpose of ministering to the sick poor only, there is no reason why such patients should not be received in the Home, but there are many objections to the plan, which we are afraid is becoming somewhat common, of using the money earned by nurses by attending upon paying patients to help in supporting the charity of district work.

### A DISTRICT NURSE FOR MERE.

Thanks to the generosity of the Duchess of Somerset, a district nurse is to be provided for Mere and Maiden Bradley. At a meeting of the Mere Board of Guardians the Duchess, who is a member of the Board, said that she would be pleased to pay £30 towards obtaining a district nurse, as she knew that there were sometimes urgent cases where life was lost because the services of a nurse could not be procured. A committee was appointed to deal with the question and report to a future meeting.

### Lectures to Nurses on Medical Nursing.

By Eveline A. Cargill, M.D.Brux., L.R.C.P.&S.Ed.

### LECTURE VI.—DISEASES OF THE LARYNX AND TRACHEA.

Diseases of the larynx give rise to difficulty of breathing on account of the narrowing of the space between the vocal cords, and to alteration in the voice, either hoarseness, loss of voice, or stridor. This may be caused by inflammation or by spasm. All acute inflammations of the larynx are dangerous, as suffocation may ensue, and especially so in children. The inflammation may be simple, membranous, or œdematous. Simple laryngitis occurs in some people whenever they catch cold, and though the symptoms are distressing they usually subside in a few days; in children the affection is more alarming, the attack of "spurious croup" often comes on quite suddenly in the night, the child awakes in terror with a sharp "barking" cough, followed by a long, loud "crowing" inspiration, the face is pale and livid, and suffocation seems imminent. The condition is generally relieved without special treatment, but a hot sponge to the throat will cut short the attack, and an emetic of a teaspoonful of ipecacuanha wine, repeated in ten minutes till vomiting occurs, will prevent an immediate recurrence. The child must be kept in an even temperature of 65°, and the air moistened by a steam kettle; he should be kept as quiet as possible and soothed in every way, as any use of the voice, especially crying, irritates the larynx.

Œdema of the larynx is very serious; it consists of an effusion of serum beneath the mucous membrane of the larynx, which may be swollen enormously and the air-passage obliterated. It comes on very quickly, generally in the course of some acute illness, and the danger is very great; surgical treatment is often required. Difficulty in breathing and in swallowing, loss of voice, and stridor are urgent symptoms.

By far the most common cause of membranous laryngitis is diphtheria. Diphtheria is an infectious disease, in the course of which inflammation of the throat occurs with the formation of a false membrane, and this membrane may spread downwards into the air-passages and is extremely dangerous when it reaches the larynx, because here the opening between the vocal cords is so narrow that it may easily be filled up and suffocation ensue. A false membrane may also form in the throat from injury, such as drinking boiling water or chemicals; but the theory that there is a "membranous croup" apart from diphtheria is not held by many authorities now, and as bacteriological methods of examination are more generally used, it is becoming more and more evident that the formation of false membrane in the air-passages (not due to direct injury) is always really diphtheritic. This is important as bearing on the question of isolation.

Diphtheria may attack persons of all ages, and even in adults may cause suffocation, but it is infinitely more dangerous in childhood. Children are very liable to this infection; their air-passages being small, are very easily filled up, and their chests are so soft and yielding, owing to the ribs being largely composed of cartilage and not bone, that they have not the resisting power of an adult whose chest wall is of firm bone. It is owing to this last cause that all chest affections in children are so serious, because they cannot use their respiratory muscles to the best advantage.

Of late years diphtheria has been successfully treated by hypodermic injections of diphtheria antitoxin, and the mortality of the disease has been very largely reduced. Antitoxin is obtained from the blood of an animal (horse) which has been given repeated injections of fluid prepared from the diphtheria bacillus until the animal is rendered insusceptible or immune to the poison of diphtheria. Its blood now contains a substance (antitoxin) which, when injected into the human body, is antagonistic to the development of the diphtheria bacillus, and so cuts short the disease.

When suffocation threatens, tracheotomy may have to be performed: that is making an opening into the trachea below the larynx, and inserting a tube through which air can reach the lungs without having to pass the obstructed part. Tracheotomy does not affect the course of the disease, but only relieves the patient from the risk of suffocation; and as death by suffocation is a terrible death, it is best to do the operation early, before the patient has suffered the horrors of threatened suffocation. The operation in itself is practically free from danger, and even if death should occur afterwards it will be easier, and almost painless. In diphtheria the system is saturated with the poison of the disease, and this may cause death apart from any throat trouble.

In nursing a case of diphtheria it must be remembered that it is a virulently infectious disease, and therefore isolation must be strictly enforced, and that the infection is mainly conveyed by means of the discharges from the mucous membrane of the air passages. The false membrane itself is swarming with bacteria, and is of extreme virulence. General directions as to infectious diseases will be dealt with in the lecture on that subject, and here I would only emphasise that the special danger in diphtheria is through the discharges from nose and mouth, therefore the nurse should avoid putting her face in front of the child's mouth, and should never let him cough in her face. If local applications have to be made to the throat by the nurse, she should protect her mouth and nose by a mask of antiseptic gauze, as a child almost invariably coughs and chokes in the process. Kissing a child with diphtheria is very dangerous.

In nursing a tracheotomy case the bed should be protected by a tent (or screens in a private house), with steam kettle going night and day to keep the air warm and moist at a temperature of 65°, which must never be allowed to drop. The nurse must watch the tube constantly and clear away any mucus with a feather, the inner tube should be frequently removed and cleaned, and the outer one cleaned out inside. If the outer tube should come out the nurse must not try to replace it but should keep the opening potent with dressing forceps till help can be obtained.

Tracheotomy may be performed for any disease of the throat which involves obstruction of the upper air-passages and not only in diphtheria, but it always requires the same careful nursing, and the supply of warm moist air at an even temperature.

There is a serious form of spasmodic croup, laryngismus stridulus, which occurs among ill-nourished children, and which sometimes ends fatally. It comes on by day as well as by night. The child becomes rigid and blue, without breathing, the chest fixed, the head thrown back, and the face-muscles twitching; then comes a long "crowing" inspiration and finishes the attack for the time. In a more severe attack there may be general convulsions and death. The fit may be arrested by placing the child in a warm bath, with a cold sponge on the head; if the child is very rigid the body should be slapped with a wet towel while the bath is being prepared, and smelling-salts applied to the nose. Artificial respiration may be used if natural breathing is long delayed.

### Cholera in Egypt.

BY A NURSE IN ALEXANDRIA.

THE cholera epidemic in Egypt is naturally arousing great interest, and numerous theories have arisen as to how the disease has once more gained admittance into the country. There is no doubt that cholera raged this year amongst the pilgrims at Mecca, and investigations have shown that the first cases in Egypt occurred amongst pilgrims after their return to their homes at Moosha. It has been found that a feast was held in honour of their arrival. The first victims were attacked the day after the feast, at which probably the *pièces de résistance* would be, as is customary, dates and zamzam (holy water) which the magic of "backsheesh" had smuggled through from the holy places. With much difficulty, the fact has been elicited that several of this group of pilgrims were attacked while at Mecca with an acute form of diarrhœa and vomiting. Although they recovered, they would still be a great source of infection to others.

### THE BEGINNING OF THE EPIDEMIC.

Moosha is a village of about 8,000 inhabitants, situated some considerable distance from the right bank of the Nile, about 10 kilometres from Assiout, and depending solely for its water supply upon a number of wells, the principal one being in the main street, and directly connected with the drain of the adjoining mosque. The natural position of this village, lying as it does, off the high road, in a valley, and best of all, away from the river bank, is most favourable for isolation; but, unfortunately, as will be seen, it was not surrounded by a cordon, until some 1,500 people had fled in various directions over the country. The physician of the markaz of Assiout, a native, has to inspect 30 villages, and can only do four, five, or six every month, as they lie long distances apart. The population of his district is 76,763, and he shares besides the work in Assiout itself, with another doctor. The population of Assiout is 42,000. The invariable rule in all districts is to inspect pilgrims as soon as possible after their return. This was done at Moosha, and reported all well on May 30th. The Omdeh, the head-man of the village, hushed up the beginning of the epidemic. It is a well-known fact that the natives, especially the country people, have a great objection to hospitals, or to being treated for disease in any but their own way. They fear that they may be made subjects for experiments, and, moreover, the Koran proscribes post-mortem examination. This omdeh was the chief of the pilgrims returning to Moosha, so his position made it easy, and his inclination as a Mohammedan prompted him to conceal the facts of the case, and he managed to do so as late as July 12th. According to statistics, the normal death-rate of Moosha per month is from 26 to 30, but during the time that cholera existed unknown to the sanitary authorities, returns sent in were less than normal, and investigations have shown a very considerable discrepancy.

### THE INFECTED WELLS.

The doctor of the markaz announced "cholera" on July 15th, but the Government delayed, for bacteriological confirmation, the official announcement till July 18th, and on July 19th a cordon was formed preventing anyone leaving or entering the place. The wells, having become infected, were stopped up, the only available water being supplied by newly bored artesian wells. The usual sanitary regulations were carried out as to whitewashing houses, baking or burning infected clothes, bedding, etc., destroying dirty zeers and goolahs (water vessels). The sick, and, as far as possible, all who had come into contact with them, were isolated. The disease has taken such a virulent form, and runs such a rapid course, very often proving fatal within a few hours, that but little can be done for the victims.

### THE DISEASE IN CAIRO.

The epidemic next appeared in Bonlac, a very filthy quarter of Cairo, the infection being imported by a woman from Moosha, who had fled to Cairo before the cordon was established. Although the sanitary authorities redoubled their energies other quarters of the town became infected, in spite of every precaution, and the number of deaths per day varied from one to eighty. The list of infected towns and villages increased until, at present, cholera has cropped up over practically the whole of the Delta.

### THE POLLUTION OF THE NILE.

It was hoped at one time that the disease could be circumscribed, but once the Nile was contaminated the only remaining precaution was to prevent further pollution. The banks of the river and canals passing through towns and villages are being watched by armed guards, with strict orders to prevent boats from mooring within a certain radius, and also to prevent persons from washing themselves, their clothes, or their animals directly in the stream. A system of pure water supply, by means of pumps, has been generally adopted throughout the country.

### THE PRECAUTIONS.

Amongst the numerous precautions which have been taken I may note the prohibition of all moulids (fairs), the closing of summer café concerts, concerts by regimental bands discontinued, no recruiting of soldiers in the province of Assiout, and waste lands enclosed by railings. The saggas (water sellers) are only allowed to supply purified water from carts which have been sterilised by steam. All the European soldiers have been confined to barracks, and native soldiers only allowed to visit their homes once a week. On the trains a carriage is reserved for the isolation of suspicious cases, and doctors with the necessary remedies are in attendance at the principal stations. People who have travelled in Egypt would appreciate the fact that nothing but purified water is obtainable *en route*.

### PROVISION FOR EUROPEANS.

In Cairo a hospital has been specially arranged for the reception of Europeans, in charge of European nurses, and patients may be attended there by their own doctors. The Greek, German, and Government hospitals in Alexandria have made arrangements to receive paying patients. In all other towns of any size a native hospital exists. If people prefer it they can be nursed in their own homes, that is if the house is found to be thoroughly sanitary and airy; but in such an event the household remains in complete isolation and all the usual precautions are taken. The poor are, if not *in extremis*, moved to the native hospital or to the one specially set apart for cholera patients. The water supply and drainage is of course specially looked after, and remedies are provided gratis to the poor by the Government doctors; but statistics show a large number of cases found dead, a dangerous factor in the spread of the malady. Notices in various languages are posted up by the sanitary authorities warning the public against errors in diet. Milk and water must be boiled before drinking, no uncooked fruit or vegetables must be eaten, meat and fish must be used only when perfectly fresh. Washing of hands before meals is advised,

CHOLERA IN EGYPT.—*Continued.*

and persons who eat with the fingers should soak them in a disinfectant. Those who know the habits of the natives, even of the higher classes, will not consider the last precaution superfluous.

### The Nursing of Cholera.

There is very little chance of nursing the sufferers, as they so often die a few hours after the beginning of the attack. The percentage of deaths up to date (August 26th) is over 88. During the first week of the illness artificial serum is subcutaneously injected, stimulants given, and of course fluid diet. If the patient's strength holds out through the acute stage, the malady generally follows a course closely resembling typhoid, and is treated accordingly. Convalescence is slow, and patients must be kept under observation until bacteriological examination proves that they are free from cholera microbes and therefore no longer dangerous to other members of the community.

### Suicide of an Assistant Superintendent Nurse at Crumpsall Workhouse Infirmary.

A painful case of suicide has occurred at Crumpsall Workhouse Infirmary. On Saturday evening Miss H. H. Asgodby, assistant superintendent of nurses, was found in her bedroom with a handkerchief tied round her throat. On Monday an investigation took place before the Coroner at the Minshull Street Court, and the following evidence was given:—

Miss Girdlestone, lady superintendent of the workhouse infirmary, said that she found the deceased stretched face downwards on the floor close to the bed. She despatched a servant for a doctor, and then tried to lift the nurse on to her bed. Unable to do this she turned her on to her back and put a pillow under her head. She then noticed a silk handkerchief tied loosely round her throat. On removing this, she discovered another handkerchief tied very tightly, and the deceased's face was very much swollen and discoloured. The doctor, who had by this time arrived, tried artificial respiration, but without any result. Witness had to cut the handkerchief in order to remove it.

Replying to the coroner, Miss Girdlestone said that so far as she was aware deceased had no trouble on her mind. She was a most efficient and experienced nurse, and was usually of a cheerful disposition.

Dr. Lempriere, resident medical officer at Crumpsall Workhouse Infirmary, said that in his opinion the deceased had been dead an hour and a half when he was summoned.

A Juror inquired whether the deceased would have sufficient strength to tie the handkerchief tightly round her throat, and then to put the other handkerchief over it?

Witness: I think that probably she tied the outside handkerchief first loosely, and then tied the other underneath.

Witness went on to say that he had known deceased 18 months. She was subject to occasional fits of slight depression, which had become rather more marked during the last four months.

Major Ballantine, Master of Crumpsall Workhouse, expressed on behalf of himself and the whole of the infirmary staff, the deep sorrow that was felt in the institution at the sad event. Deceased was a lady of very high character, and her untimely end had come as a very great shock to everyone who knew her.

The Deputy Coroner (Mr. Aitken): It is extremely sad. Have you noticed anything singular about her lately?

Major Ballantine: I had noticed a slight irritability of late. Little things seemed to worry her which did not affect her before.

The Coroner: She was a most efficient lady, I believe? Most efficient in every way.

Mrs. Elizabeth Thornton, of Hazel Grove, said that deceased was her sister, but she was not aware she had any trouble. She had not shown any signs of mental aberration, and a fortnight before her death witness received a letter from her written in her usual manner.

Miss Girdlestone mentioned that the deceased made a kind of will a fortnight before her death. It was not formally witnessed, but contained a few directions as to the disposal of her property. No letter or document of any description was found hinting at intended suicide.

The Coroner remarked that the case was a very sad one, and he was sure they all sympathised very deeply with the relations of the deceased. There was very little doubt, though there was no direct evidence on the point, that the unfortunate lady was out of her mind when this occurred.

A verdict of suicide whilst temporarily insane was returned.

### Novelties for Nurses.

By Our Shopping Correspondent.

### "LINETTA" COLLARS AND CUFFS.

"Linetta" collars and cuffs are a most useful addition to a nurse's outfit. It is difficult to believe that these articles of apparently excellent quality linen are manufactured of paper, and certainly no one would detect the fact without closely inspecting them. It must be a boon to nurses who are moving about not to have to depend on the laundress, and also to save some expense, for "Linetta" specialities cost less than the price of washing. The collars and cuffs are obtainable at all drapers. They are made in various styles; those named "Ambulance" are especially suitable for nurses' use.

### HYGIENIC UNDERCLOTHING.

Now is the time to replenish one's stock of warm underclothing, for the constant changes in the autumn temperature is especially dangerous to the lightly clad. I recommend to the attention of nurses Dr. Thomballa's double-texture underclothing as very suitable under the circumstances. It is soft, light, and warm, and more comfortable and durable than a material made solely of wool. It is a mixture of raw cotton and wool. The manufacturers claim for it that the inner layer of cotton does not absorb the perspiration and retain it next the skin, but that it allows it to pass freely to the outer and more absorbent surface of wool, thus preventing the unpleasant sensation of dampness which woollen garments give when the body is heated by exercise. The material does not shrink, is moderate in price, and the garments are well made. They can be obtained through linendrapers, or of Messrs. Zimmerli and Handschin, 65 Wood Street, London, E.C.

### The Silent Hour in a Children's Ward.

BY A SISTER.

"Habits grow by unseen degrees,
As brooks run to rivers, rivers run to seas."

My personal experience has taught me that it would be scarcely possible to choose a happier lot for a nurse who has a knowledge of, and love for, children than to be appointed sister of a children's ward. When I was reading in The Hospital of August 30th about "A Silent Hour," I was reminded of the days, now some way back in my nursing life, in fact before that life commenced at all.

### The Value of Kindergarten Training.

I had spent two years in a Frœbel Training School learning the Kindergarten system, and, after teaching for a few years, I gave up the work in favour of nursing sick children. Those who have nursed in a children's ward, and found the endless need of patience, tact, and resource in themselves, will realise how very much the one training helped the other. When I was given my own ward, and the children became as it were my own, to plan for, and to help through some of the weary hours of the day, I saw the possibility of making use of some of the kindergarten work, for there are so many stages in sickness when half the battle is to find occupation and to create a happy, light heart, if possible. I commenced a systematic use of the "Kindergarten Occupations," and it was really lovely to see how quickly the children took to them, and how eagerly they looked forward to them from day to day.

### "Our Methods."

We worked it in this way. As soon as the ward was tidy, the medicines given round and the hands and faces washed, the "silent hour" began—and while the children slept or lay quietly, I slipped into my room and prepared the work, threading the needles, correcting little mistakes, preparing a new design, or modelling an object in clay, to be copied. The nurses in the hospital had provided me with collar or boot boxes—each of which had a clean piece of paper at the bottom and the child's name written on the lid—and these boxes contained whatever they were working upon at the time, with their mat-needle, pricker, pencil, paint-brush, etc. We went in for mat-plaiting, pricking objects, paper folding and cutting, drawing, painting, and modelling in clay, chosen according to whether the children were sitting up or lying down, or up and dressed, and able to sit at the table. At half-past two I went round with the boxes, always making use of the elder children who were convalescent, as my satellites, and they were very helpful in starting the work, and "keeping the ball rolling."

### The Question of Time.

Now I know that many hard-worked sisters and nurses would think it was not possible to find the time, but I must explain that we only indulged in this enjoyment three days in the week, and I never kept to any special days, so that if a bad case came in, or if I had any operation cases, or one of my nurses "off duty" for her "long day" my work-boxes never appeared at all; yet there were many days when we worked away steadily for an hour and a half. My probationers would often help if they had a few minutes to spare, but as a rule they were busy cutting bread and butter for tea, padding splints or attending to the little ones, who were too ill or too small for any occupation at all. The elder children were allowed to keep their boxes out until bed-time; and several little girls who were handy with their needle and thread, soon learnt to make up the younger children's work into useful and pretty trays, pin-cushions, hair-tidies, etc. There was a male medical ward on the same floor as my children's ward, and I remember with pride sister coming into my room one day at tea-time, saying "My men want to know what has happened to the children, for they say that they never used to have such undisturbed afternoon naps, until you came to be sister up here."

### "Show Day."

It happened to be visiting day, which we always made our "Show Day"—spreading out our work on the table and bed-boards, or hanging it upon the sides of the cots; so when I took the medical sister round and showed her what the children had really accomplished in the few weeks they had been at work, she was most appreciative, and went back to explain to the men the reason of all the quietness on the opposite side. I often found it would comfort a little home-sick body if I could promise a fresh pretty piece of work to show mother when she came again. It was always a happy subject for chatter during the process of a bad dressing in the morning, and very often quite sufficient punishment, to withhold the work altogether, if some little person had been troublesome, disobedient, or quarrelsome, and refused to be called to order.

### A Hint to Nurses in Embryo.

Of course there is principle as well as practice to be learnt in Frœbel's Kindergarten system; and frequently when I read in "Notes and Queries" of The Hospital of so many girls only 18 or 19 asking the Editor if they are too young to commence hospital training, I long to suggest that they should study Frœbel's method of cultivating happiness and intelligence in children by going for a year or two to one of the training schools. They would there learn the real meaning of his simple but grand motto, "Come, let us live for our children," and in the end would assuredly find themselves endowed with greater fitness and power to enter the large London hospitals to commence their training as nurses.

### Presentations.

Rugeley District Hospital.—Miss Hannah F. Philbrick, Lady Superintendent of the Rugeley District Hospital, has retired, having held that post for upwards of 17 years. At the committee meeting on Thursday last week the Rev. Edward Samson, in the absence of the chairman, presented Miss Philbrick with an illuminated address and a cheque for £69 13s. 3d., amount contributed by patients and others. In making the presentation Mr. Samson spoke in very high terms of Miss Philbrick's ability, and referred to the great loss which the committee had sustained by her retirement. He gave her the best thanks of the committee for her good management of the hospital, her kindness to the patients, and courtesy to all, whereby she had always maintained a high state of efficiency and good order and earned the regard of all. Miss Philbrick feelingly replied, thanking the committee for their great kindness towards her.

### To Nurses.

We invite contributions from any of our readers, and shall be glad to pay for "Notes on News from the Nursing World," or for articles describing nursing experiences, or dealing with any nursing question from an original point of view. The minimum payment for contributions is 5s., but we welcome interesting contributions of a column, or a page, in length. It may be added that notices of appointments, entertainments, presentations, and deaths are not paid for, but that we are always glad to receive them. All rejected manuscripts are returned in due course, and all payments for manuscripts used are made as early as possible after the beginning of each quarter.

### Beyond the Seas: Six Months in Tierra Del Fuego.

BY AN ENGLISH NURSE.

WHAT is Tierra del Fuego like, and do you ever want to go back to South America again? are two questions I have been asked innumerable times since I returned from Chile. If Tierra del Fuego—whither I went to nurse a lady in her first confinement—were the only spot in South America, I should say decidedly "No;" but having passed through the harbour and seen Rio Janeiro like a vast flower garden under an unclouded sky, one knows that the Argentine flag flies over many a spot of tropical splendour which once seen would make one long to return again and again. But even viewed from the deck of the mail steamer, Tierra del Fuego is cold and barren looking. It was the sun shining on the snow mountains which gave the discoverers the mistaken idea of volcanic eruptions, hence the name Tierra del Fuego, *i.e.*, land of fire. One's first impressions are right in spite of English hospitality. My most vivid recollection of six months passed on the island is an incessant wind and a dreary waste of sundried grass to look at. There are but few settlers, and they are all engaged in sheep-farming. The farms, or estancias as they are called, are large tracts of land divided into vast camps where sheep by thousands graze, and are left to themselves day after day. Porvenir is the chief landing-place in Tierra del Fuego, and it is to that small township that the mails are sent from the mainland. It is there also that the doctor, the only one on the island, has his headquarters. Porvenir is in telephonic communication with the nearer farms.

### A Memorable Ride.

I embarked at Liverpool and arrived at Punta Arenas four weeks later, and I then crossed over to the island, the passage occupying five hours, and after eight hours' rough riding arrived at Useless Bay settlement. A settlement consists of several corrugated iron buildings and the settler's house. There I was received with great hospitality, offered the usual American "cocktail," and entertained as a matter of course. Leaving Useless Bay the next day I, with two other riders, rode 40 miles along a grass plain without a tree, and varied only by huge boulders of granite, vast lagoons, the cries of the wild birds, the distant bleating of sheep, the occasional glimpse of foxes, the guanacoes feeding at a distance, and the wild cattle outlined against the horizon. In all that 40 to 50 mile ride we met no human being, and only saw one shepherd's shanty some distance from the tract, which is ploughed and fairly well trodden down by shepherds, riders, and the bullock carts carrying tinned provisions from the port, or wood from the neighbouring monte (forest) to make or repair the fencing and gates, the former being erected from slabs of wood and barbed wire which is sent out from England, as everything is.

### The Settlement.

Arrived at my destination, which is arranged much like Useless Bay settlement, only that Nature has been kinder, so that instead of the unbroken plain the Carmen Sylvia mountains lie to the north some 40 miles distant, and lend a picturesque background to the red-roofed settlement. It being October my host was busy superintending the shearing, dipping, and drafting of the sheep, and after that the branding of the year's stock with the farm's distinctive mark. By May the work is over, the wool bales are shipped, the hay gathered in, and the settlers are preparing for long months of cold and isolation, as during the winter horses are too "poor" to ride and the roads nearly intractable, due to the rivers overflowing, the heavy snows, and the perpetual frosts. Seven years ago the settlers lived in hourly dread of raids from the Indians, a copper-coloured race of fine physique but low mental abilities. Many are the tales of Indian atrocities, but now the few at large are on friendly terms, whilst numbers have been sent to Dawson Island, the Protestant mission, or Rio Grande, the Roman Catholic. The idea of being sent to either of the missions is looked upon with horror by the Indians at large. To them to go to Rio Grande or Dawson Island means to die, and the tremendous mortality, in spite of care, lends itself to their superstitious fear. Out of over 100 Indian women and men at Rio Grande 90 per cent. showed signs of tubercle bacilli either in an advanced or incipient stage, mostly the former, as tubercle once attacking an Indian seems to make appalling strides. The housing of the Indians at both missions is everything that can be desired from a sanitary point of view, but the race seems unable to bear civilisation, and, like the Red Indians of North America, is rapidly approaching extinction.

### Death in an Indian Camp.

My first sight of an Indian camp was in connection with sickness. Early one Sunday morning an Indian, wearing only a guanaco skin, came to the "big house," and through the medium of an Indian who had been some time with the settlers we gathered from him that an Indian was muche infirme (very ill), and that his great wish was to die in the settlement. Without delay we rode in the direction indicated by the smoke arising from the camp fire. In a valley on the range we came on the encampment, a dilapidated tent, probably begged from the settlers, women, children and men gesticulating and uttering weird sounds wholly unintelligible to us, and in their midst, propped up against one of the tent poles, was the sick man. One glance was enough to tell us that he was past human aid, even if one of the world's greatest physicians had stood by him instead of his tribe "medicine man." The sick man having passed away as the Indians hold to a god who lives under the earth, we improvised a stretcher, and after much expostulation prevailed on the Indians, in spite of their superstitious dread of the dead, to carry him down to the settlement, where he was buried with due reverence. Many of the settlers have Indians as servants, but they are not satisfactory; the nomad instinct is so strong that no sense of gratitude will keep them from moving on sooner or later to another settlement or returning to their tribe. Each tribe has its doctor, who uses herbs medicinally and practises a kind of massage. However, the Indians are fast losing faith in them, and pass the tale from tribe to tribe how the white doctor can cure disease and heal the broken limb.

### A Surgical Paradise.

Five years ago there was no doctor on the island, the settlers prescribing for their own labourers' wives and children; but now the English doctor is continually journeying from settlement to settlement, and when his services are required it is customary to send riders in opposite directions, but long and weary is the waiting for him, for even should he be at the next settlement nearly 24 hours must elapse before there is any hope of seeing him. Luckily, the island is healthy, a surgical paradise in fact, the war between aseptics and septics being practically unknown. An amputation case I nursed, performed with the fewest possible instruments and without antiseptics, healed by first intent. A Chilian labourer, who had received a severe knife wound, the intestines protruding through the aperture, in spite of lying in his working clothes—none too clean—and the

### BEYOND THE SEAS: SIX MONTHS IN TIERRA DEL FUEGO.—*Continued.*

wound exposed to the air, made an uninterrupted recovery on his wound being duly attended to. Indeed it is marvellous what splendid results are obtained in any cases requiring surgical attention. But I have heard of numbers of cases in which a doctor's services would have saved life, and doubtless there will be many others, for it is impossible for one man, his practice extending over such a vast area, to be just where his services are most required at a given time. When one thinks of the difficulty of obtaining medical assistance, added to the many other hardships of a life in Tierra del Fuego, one wonders how anyone can leave the beaten tracks of men and settle under such an inclement sky. I, for one, after my first experience, would not again face, among other disadvantages, the incessant wind and the intense cold.

### District Training under Queen Victoria's Jubilee Institute.

BY A DISTRICT NURSE.

HOSPITAL training finished, a nurse begins to wonder what step she will take next. She debates within herself the question of remaining in hospital or the advisability of choosing private or district work.

I decided on the latter branch, and will describe briefly the training. The necessary application being made and accepted, I was sent by the institute to —— for district training. I arrived at the home after a long journey, and was kindly received by the superintendent. I was given some refreshment, after which I was taken to my room, where I had a good rest and did my unpacking. I made the acquaintance of the nurses at supper. The meal was a pleasant one. I found the nurses agreeable, and was struck with the homelike appearance presented. The nurses seemed happy and at ease; there was an absence of constraint. Next morning I was introduced to what was to form my work during training. Having got a district bag and the necessary outfit, I was accompanied by the superintendent, who explained the nature of each case, at the same time instructing me how work was required to be done under the Queen's Institute. Even after hospital training one feels at first very much at a disadvantage when required to nurse a bad case in a cottage. With teaching from one experienced in the work the feeling passes off, and a method of working is acquired suited to the circumstances.

### THE SUPERINTENDENT AND THE PATIENTS.

During training the superintendent went round regularly. She not only explained the method of nursing in district work, but used every opportunity which arose during the morning's round, to instruct her pupil, not only for her present help, but to serve in her future work, when required perhaps to act at once on her own judgment. Her interest extended to the friends of the patient. Negligent mothers were judiciously taught many little things. An infant with ophthalmia or thrush, was never passed by; a cut finger, or any simple accident was attended to, and the occurrences used as object lessons to the mothers. The surroundings of the patients were duly regarded, and suggestions made for their improvement when necessary. The sanitary arrangements were always well looked into. The people were encouraged to help themselves. Their co-operation with little things for the comfort of the patients was often solicited for that object. Conversation was directed to arouse their interest in things outside their daily work. Papers and periodicals were frequently lent with the same view.

### IN THE HOME.

Our days in the home passed with clock-like regularity. Called at 7 A.M. by the housemaid who brought each nurse a can of hot water, breakfast was served at 7.45, after which we each made our own bed and prepared for going on the district. We went out at 8.30. Dinner was at 1.30, for which meal we were generally in, and always ready. We had tea at 4 o'clock, and started again at 4.30 for our evening round. We got done about 7, occasionally a little sooner. After supper, we gave to the superintendent the report of our work and time on duty with the number of visits paid. We then renewed our bags, and so ended the day.

### INSTRUCTION.

During training we had lectures on the following subjects:—"Monthly nursing, and the diseases of women," "hygiene," and "fever nursing." There was also a course of instruction on "district nursing," the working of a district, and the appliances and stock required by a nurse. The course ended by a demonstration of sick-room cookery. These lectures were given in the afternoons, generally two a week. The remaining afternoons—Saturday excepted—from 3 to 4 o'clock, we spent in preparing our studies. We wrote notes on the lectures and at the end of the course had an examination. The acute cases were visited, when necessary, twice daily. On Sundays only patients requiring special attention were seen to. The work was interesting, we were fully occupied, and the time of training passed by quickly and pleasantly.

### Everybody's Opinion.

**[Correspondence on all subjects is invited, but we cannot in any way be responsible for the opinions expressed by our correspondents. No communication can be entertained if the name and address of the correspondent are not given as a guarantee of good faith, but not necessarily for publication. All correspondents should write on one side of the paper only.]**

### "AN AWFUL PLIGHT."

"G. S." writes: I was intensely amused a few days ago when I was at a tea party to hear some ladies who had been visiting one of my old patients in a Cornish village tell the following story. The patient had had enteric. When the village folk heard it was typhoid fever, not a soul would go near to help for love or money. The doctor, knowing this, suggested that I should come from the neighbouring town, and show the wife how to change the bed, shirts, etc. This I did, and, of course, I had the shirts opened and put on pinafore fashion. Even now—the patient is quite well—he thinks it was a mistake, and delights in telling everybody that he was in an "Awful plight." Sure enough, he couldn't put on his shirt himself, and his wife couldn't put it on, so the doctor had to send a young woman from the town to show his wife, an old married woman, how to put it on, and then, if "she didn't go and put it on the wrong way!"

### THE LIGHT SIDE OF DISTRICT NURSING.

"PING PONG" writes:—After reading "F. P.'s" letter in THE HOSPITAL, I thought that the following experience of mine might perhaps interest some of your readers. A short time ago I was nursing the little daughter of a personal friend at whose house I naturally felt more free, *re* visits to kitchen, etc., and was one morning busy shredding beef for making beef juice, when the butcher's man, after a loud knock with a riding crop, walked in. I replied to his cheery "Good morning, miss," and also volunteered the information that "cook would be back presently." Without waiting for any further invitation, he asked if I found my place comfortable, and started to tell me that "cook was a good old sort, the missus a bit particular 'bout the meat, but not bad to get on wi'. The church was two mile away, he belonged to the choir and always went regular Sunday nights. 'Twas terrible quiet at S——, didn't think he should stay long." I left by one door as cook appeared at the other, and later "that good old sort" told me I had been pronounced a tidy looking bit, but very quiet and sort a stuck up. "I told him you was a special friend of the missus, and had come to nurse Miss Nellie. He went off with his gert rid face a site ridder. The hignerance of these country louts is hawful," continued cook. The fact was he had never seen a nurse, and mistook me for a new kitchenmaid, who, like myself, had arrived a few days before.

### THREE MONTHS' MIDWIFERY TRAINING.

"H." writes: In her article on "Three Months' Midwifery Training" one of your contributors speaks of there being little time for practice. How can a nurse learn all the little details of general nursing in so short a time? Even if a woman is painstaking and anxious to do what she can towards alleviating pain and discomfort she cannot know how to go about it like one who has had more experience I was sent from the institution to which I belong to a lady who had been confined nearly three weeks. I found the temperature nearly 104°, and fomentations had been ordered by the medical man. A flannel apron was put on the abdomen with a large mackintosh above it to keep in the heat; while the food, which was not being digested, was steadily given, despite the fact that the patient complained of general discomfort and pain after cold milk. Then again, although there had been high temperature for two weeks, there had been no sponging done, excepting hands and face, since the child was born; consequently there was no sleep until three or four in the morning. The infant, who was hand-fed, had been so mismanaged that at the end of the month a highly-trained nurse had to look after him alone.

### THE HOME SISTER'S AUTHORITY.

"Another Home Sister" writes: I quite agree with the Home Sister of a Liverpool Institution about whom a nurse wrote on August 30th. As the head of an institution I think her wishes should be consulted, perhaps more so as no uniform is worn. I think, also, that sailor hats are not suitable for Sunday wear, but this, of course, is a matter of taste. Still, if "Sister" felt her nurses were anxious to carry out her wishes with regard to their dress, she would meet them half way. As to stopping a nurse while with her friends to ask when her boots were last cleaned, I can only conclude that if it annoyed the nurse to be asked such a question, it must have much more annoyed the "Sister" to see any of her nurses in boots that needed such a remark. May I close with a few words I heard the other day? Two nurses were going out from an institution not in uniform, and I heard someone who saw them say: "Fancy being ill and having a nurse to attend one like either of these two girls." I was sorry it should be said, but I was not surprised, for they were truly untidy and down at heel. I wondered what the matron of the institution would have thought had she heard the remark.

"Sailor Hat" writes: With reference to the discussion *re* "Home Sisters" and their authority, I believe nurses, on the whole, fully understand the difficulties of the position, and, with all due deference to "Nurse Elizabeth," it is the minority and not the majority of nurses who would so far forget their natural politeness as to accord to the "Home Sister" the treatment of an upper servant. At the same time I think a less public and more fitting time and place might be chosen for the pointing out of "dirty boots," etc., than the public thoroughfare. The question of wearing "sailor hats" on Sunday where "bonnets" are forbidden is beneath notice. What is more neat or becoming than a sailor hat? Why should nurses in "training"—especially those who intend to follow private nursing—burden themselves with fashionable hats and cause the hospital they are connected with to be called "a Sunday millinery parade." If the matrons of our large infirmaries and hospitals would, instead of forbidding outdoor uniform on Sundays, unite together and procure a recognised—registered if need be—uniform, with the power to fine any unauthorised person wearing an imitation of the same, the hat difficulty would be settled for aye. I have known nurses, where outdoor uniform is forbidden, remain indoors on Sundays, neglecting their church or chapel, all because they had only the despised "sailor" and could not compete with their more fortunate sisters in fashionable attire. The great objection to outdoor uniform amongst nurses is, I believe, the constant stream of nursemaids and bassinettes one meets. Here the fault lies with the mistress, not the maid. This trouble a recognised uniform would effectually prevent.

"A Late Liverpool Nurse" writes: In my opinion, outdoor uniform not being worn nor provided for the nurses in a certain institution in Liverpool, the home sister has no authority at all to say whether the nurses shall or shall not wear a sailor hat on Sundays, be the Sundays wet or fine. Some women who go to train for nurses have no money except the small salary given them whilst training, and therefore have to be very careful of their hats. I was trained at an institution in Liverpool, not, of course, necessarily the same as the one mentioned in your issue of August 30th, and "the sailor hat on wet Sundays question" often arose whilst I was there. Neither then nor at any time since have I been able to see what right the home sister, or anyone else, had to interfere with the nurses' private clothes worn outside the institution, the agreement being for them to wear no outdoor uniform. Referring to the home sister stopping the nurse in the street, and asking her when she cleaned her boots last, I may say that this is a course of action most strikingly characteristic of the home sister where I was recently trained. Is it, may I ask, dignified for anyone occupying the office of home sister, and ranking next in position to the matron, in a large institution of nurses, to openly and frequently lose her temper, and repeatedly say to them for some trifling fault or other, such as upsetting a cup of tea, or omitting to replace the sitting-room chairs against the wall, that "she does not know what kind of homes they have been brought up in?" Can the nurses have any respect for the home sister who takes a very evident delight in humiliating them on every possible occasion?

"Fair Play" writes: Like "Nurse Elizabeth" I was much interested in "A Liverpool Nurse's" letter, and took the trouble to make a few inquiries. I find that "A Liverpool Nurse" has been very mild indeed in her complaint, as the matter to my mind seems a case of downright despotism on the part of "Home Sister." Does "Nurse Elizabeth," who has taken up the cudgels on behalf of "Home Sister," think that nurses have not their little vanities in the way of headdress the same as other people, and does she think that out of the princely salary some of them receive in return for their arduous and very often unpleasant duties, that they can provide themselves with headgear to suit every change in our variable climate and in the mind of home sister? I quite agree with "Nurse Elizabeth" that it is the duty of each nurse "to heighten the standard of the hospital they serve by their behaviour," etc.; but how is this to be done when the only thing which identifies them with their particular hospital, viz.: uniform, is forbidden them when out of doors? Perhaps "Sister Elizabeth" can tell us whose duty it is to look after the requirements of a nurse lying sick in the home. Is this the duty of another nurse who has been on duty all night, or of the home sister, and does the latter see to this? Again, is it the duty of nurses who have been on duty either all day or night to see to the sorting of the linen from the laundry, or of the home sister, and again, does the latter do this? Further, I do not think it tends to cultivate the good feeling which should exist in an institution of this kind for any nurse to be addressed in such terms as "I wonder what kind of a home you can have come from," and yet these are the words of a home sister. I think your editorial note, that "the home Sister should be careful in the exercise of her authority to temper it with tact," hits the nail on the head.

### Appointments.

[No charge is made for announcements under this head, and we are always glad to receive, and publish, appointments. But it is essential that in all cases the school of training should be given.]

Birkenhead Union Infirmary.—Miss C. H. Crozier has been appointed charge nurse. She was trained for three years at Salford Union Infirmary.

Grove Hospital, Tooting.—Miss Alice A. Browne has been appointed matron. She was trained at the London Hospital, and has since been night sister at St. Pancras Infirmary, Dartmouth Park Hill, London, and superintendent nurse at the Union Hospital, Newcastle-on-Tyne.

Roxburgh District Asylum, Melrose, N.B. — Miss Janet Scott has been appointed head nurse and deputy matron. Miss Scott was trained at the Glasgow Western Infirmary.

Royal Albert Edward Infirmary and Dispensary, Wigan.—Miss E. E. Fletcher has been appointed assistant matron. She was trained at the Wigan Infirmary, and has since been in succession sister of women's wards, night superintendent, sister of men's surgical wards, and theatre sister.

South-Western Hospital, Stockwell, London.—Miss Lucie M. Toller has been appointed charge nurse. She was trained for three years at the Nightingale School of St. Marylebone Infirmary, Notting Hill, W., and afterwards held the appointments of special nurse at the Royal Infirmary, Edinburgh, and of sister of the medical wards of St. Bartholomew's Hospital, Rochester. She has also had experience in private nursing.

Stephen Cottage Hospital, Dufftown.—Miss Agnes Ramsay has been appointed matron. She was trained at Glasgow Western Infirmary, and has since been sister and temporary matron County of Lanark Fever Hospital, superintendent nurse at Dover Hospital, and matron of the Hatton Nursing Home, Ceylon.

Wortley Isolation Hospital, Greenside, near Sheffield.—Miss Annie Elizabeth Clack has been appointed nurse-matron. She was trained at the Royal Infirmary, Preston, and has since been charge nurse at the Park Hospital, Hither Green, London.

Wrexham Infirmary, Denbighshire.—Miss Agnes Maud Oslar has been appointed matron. She was trained at the Royal Albert Edward Infirmary, Wigan, and has been working in connection with that institution for the past seven years. She has held in succession the post of sister to men's surgical wards, night superintendent, and for the past fourteen months she has been assistant matron. Miss Oslar holds the L.O.S. certificate, and for a short time she acted as superintendent at Miss McCaul's Home in Welbeck Street, London.

### Wants and Workers.

The matron of the Cottage Hospital, St. George's, Shropshire—a poor mining district—would be glad if anyone could give her a gentleman's cast-off dressing gown, for the use of the patients.

Will anyone kindly lend a spinal carriage to a man with spinal caries who cannot afford to buy one? Address Nurse Rust, the County Hospital, Taunton.

### For Reading to the Sick.

### CLING TO ME.

Oft when I seem to tread alone
Some barren waste with thorns o'ergrown,
Thy Voice of love, in tenderest tone,
Whispers, "Still cling to Me!"

Though faith and hope awhile be tried,
I ask not, need not, aught beside:
How safe, how calm, how satisfied,
The souls that cling to Thee!

They fear not Satan or the grave,
They feel Thee near, and strong to save,
Nor fear to cross e'en Jordan's wave,
Because they cling to Thee!

Blest is my lot, whate'er befall:
What can disturb me, what appal,
While as my Rock, my Strength, my All,
Saviour, I cling to Thee?

*C. Elliott.*

Religion is, to man in his fallen state, more than what medicine, fresh air, and nourishing food are to one who is sick. It is a new life, because it makes us partakers of the eternal life of God. Religion is to human life what a chart, compass, and rudder are to a ship, or the metals and brake are to a railway train. A ship is guided, and a train is directed and controlled, by means of these things. So human life is controlled, regulated, and directed by religion in the face and presence of moral evil. "Thou wilt keep him in perfect peace, whose mind is stayed on thee"—this is the root-idea of personal religion. "Hold thou me up, and I shall be safe"—this is expression of personal religion. "Made for thee, the heart is restless until it find thee"—this was the confession of one who tried to live without God and personal religion, and who found out his mistake before it was too late.

God made man for fellowship and friendship with Himself, both here and hereafter. God claims the souls of men for His own everlasting possession. "Behold, all souls are mine." It is by the exercise of personal religion that we yield ourselves, one by one, to His claim. Religion, on man's part, is the moral effort to correspond with God's great purpose of salvation and sanctification. Religion, on God's part, is the Divine encouragement to us to do so, the gift of grace which alone gives effect to, and crowns such effort. "My son, give Me thine heart." It is in the exercise of personal religion that man offers his heart and life to God as his Father. Personal religion is the realisation of our filial relationship to God, the recognising and claiming our rightful position and true dignity as sons of God. Personal religion develops the son-like character in the face of moral evil.—*Vernon Staley.*

For there's a Land where suffering dies for ever,
A "quiet habitation," where our Lord
Will lead his chosen by a still calm river
And pastures fair—so saith th' Eternal Word.
There will the sick the great Physician meet
And healèd, rest for ever at His feet.

Then shall the eyes be opened that were dim,
And shall the King in all His beauty see.
Deaf ears shall hear th' adoring Angels' hymn,
And silent lips shall pour forth melody.
Strength to the feeble shall return for aye.
There joy shall reign and sorrow flee away.

*C. M. Prevost.*

### Echoes from the Outside World.

### The King and Queen in Scotland.

After leaving Stornoway last week, and remaining in a sheltered bay near Thurso for a day owing to the gale, the *Victoria and Albert* anchored off Dunrobin Castle, and the Duke and Duchess of Sutherland came aboard and remained for luncheon. In the afternoon the whole party went ashore for afternoon tea at the Castle. The next day the King and the Duke of Sutherland went for a deer drive, but though the King got two difficult shots there was no kill. The Queen and the Duchess joined his Majesty later at Loch Brora for lunch, and then returned to the Castle again for tea, where there were about forty guests present. On Saturday an aquatic show was organised, and both the King and Queen seemed to enjoy the novelty of the entertainment. The swimming instructor was a Swede, and he was most delighted that the Queen conversed with him in his native language. Her Majesty was very busy with her Kodak all the time. In the afternoon the King visited Mr. Carnegie at Skibo Castle, whilst the Queen inspected Lawson Cottage and drove to Little Ferry, where her Majesty took tea with Mr. Wright, the late Duke's private secretary. Their Majesties derived much gratification from being able to revive the memories of their visit to Dunrobin Castle twenty-six years ago, and their four days' stay at Golspie was an especially pleasant time. On Monday the King and Queen left for Balmoral. They landed at Invergordon Ferry Pier, drove to the railway station, and so by way of Elgin to Ballater. The route by the Moray Firth coast-line has never before been traversed by Royalty. Owing to the King's expressed wish, the decorations at Balmoral and en route were of a simple character, but the welcome accorded him by the Scotch people was very hearty.

### The President of the United States.

An accident to the American President last week fortunately resulted in no serious consequences to Mr. Roosevelt himself beyond a cut mouth, a swollen cheek, and a discoloured right eye, but one man was killed and the driver of the carriage had his skull fractured. The President and his party were being driven down one of the streets of Pittsfield, and when they came near to the County Club a motor car approached at a terrific pace, and dashed into the President's car diagonally, literally smashing it to pieces. Mr. Roosevelt and three other occupants were hurled some distance and fell in a heap together, one being taken out dead. The horses also were killed. The President rose after a few seconds and walked to the motor man of the trolley-car, who was paralysed with fear, and questioned him as to his behaviour, but he was too frightened to reply. When Mr. Roosevelt was congratulated upon his escape he said, "There are things worse than death," and afterwards repeated the same remark. The conductor and the motor man of the car have been charged with manslaughter, and it is not yet known if the affair was really an accident or part of a plot. King Edward, the German Emperor, and M. Loubet have all telegraphed congratulations and made inquiries.

### Another Disaster at Martinique.

Fresh eruptions at Martinique have done such terrible damage as almost to reduce the island to complete devastation. Morne Rouge, which had escaped the previous catastrophe has been engulfed. The loss of life is computed at over 1,000. One village, Ajoupa Bouillon, is in ruins, and 200 of the inhabitants are dead, 400 being seriously injured, many not expected to live. Both towns were overwhelmed by a river of boiling water and mud with flying stones. The sea was terribly agitated, and there was a tidal wave all along the coast. After an explosion a portion of the eastern part of the island a mile in extent sank into the sea. At St. Vincent, La Soufrière seems to have been almost as active as Mont Pelée, at Martinique, though the results are not so far-reaching nor so destructive. The volcano began at first discharging dense clouds of steam, with much internal rumbling, and those who lived in the vicinity were warned that they must leave. The next day the discharge continued all day, the sun was obscured and the heat oppressive. Sand has fallen 10 miles from the crater, but the sea is only slightly agitated. The arrow-root plantations in many places have been ruined, and the Rabacca river is a stream of fire a quarter of a mile wide. The appearance of the summit of La Soufrière has changed, and a piece of the mountain has evidently been blown away. It is not only at Martinique and St. Vincent that volcanic disturbances have taken place, for in Asia the ships sent to Torishima have returned to Japan to report that there is no trace left of the hamlet and population of 120 persons. It is not known if they were engulfed by the sea or buried by lava. A new bay has been formed, the centre of which is a boiling whirlpool, and there are no evidences of previous habitation except the carcases of two oxen.

### A Great German.

Professor Virchow, the well-known German pathologist, anthropologist, and politician, died last Friday in Berlin at the age of 81. In January of the present year, when alighting from an electric tramcar, he fell and sustained a fracture of the *cervix femoris.* The fracture was not of a complicated nature, but the Professor's advanced age made the case serious, and the bone never united properly. He had been to Harzburg for his health, but expressed a wish to return to Berlin, and thither he was brought on a stretcher a little less than a week before his death. Professor Virchow was a native of Pomerania, and as soon as he left school he applied himself to the study of medicine, devoting his energies to the scientific, rather than the practical, side of the profession. The result of his researches frequently appeared in a scientific magazine which he helped to found. He conducted it for half a century, and it is a perfect storehouse of useful information. He was employed in 1848 in investigating a severe outbreak of typhus fever in Silesia, and made a considerable stir by his sweeping denunciation of the evils he had seen. It is owing to his constant advocacy of sanitary reform that Berlin can no longer be reproached with unhealthiness, and he also did much in improving the hospitals, the water supply, and the police regulations. When the Franco-German War broke out he assisted in the organisation of the ambulance work, and to the end of his life he retained his keen interest in all matters concerning the public health. His eightieth birthday was celebrated by a world-wide demonstration of respect, and Lord Lister paid him a magnificent tribute. Professor Virchow was a man of many parts, and he excelled in all he undertook. He never judged hastily, but having made up his mind he fearlessly expressed his convictions. He did not hesitate to run counter to public or to Royal opinion, as in the case of the cure for consumption advocated by Koch, which he pronounced against on scientific grounds, being, on the contrary, enthusiastic about the anti-toxin discovery for diphtheria, which he contended it was the bounden duty of every practitioner to employ.

### Notes and Queries.

**The Editor is always willing to answer in this column, without any fee, all reasonable questions, as soon as possible.**

**But the following rules must be carefully observed:—**

**1. Every communication must be accompanied by the name and address of the writer.**

**2. The question must always bear upon nursing, directly or indirectly.**

**If an answer is required by letter a fee of half-a-crown must be enclosed with the note containing the inquiry, and we cannot undertake to forward letters addressed to correspondents making inquiries. It is therefore requested that our readers will not enclose either a stamp or a stamped envelope.**

*Nose.*

(135) 1. Will the Editor kindly say if there is any means of altering the shape of the nose? A child I know has fallen on her face, and now her nose is a veritable pug. I seem to recollect hearing of a machine for such a purpose, and shall be much obliged if you can give me further information. 2. Can you tell me where I can obtain the periodicals "Nursing Notes" and "Baby," and tell me the prices?—*M. L. R.*

1. Wonderful things are now done in plastic surgery. The best plan will be to consult a medical man. 2. "Nursing Notes" may be obtained from the Editor, 12 Buckingham Street, Strand, W.C., price 2d., and "Baby" may be obtained at the office, Agar Street, Strand, London, W.C., price 6d.

*Lupus.*

(136) I am interested in a case of lupus. The patient is a woman of 20, and the right nostril is nearly gone. Will you kindly tell me how I can help her for she is poor? Is there any way by which she could have the advantage of the Finsen light treatment, and which is the nearest place to Coventry where it is available?—*B. C. R.*

You might get the patient into a Birmingham hospital. The admission is free. Failing that, write to the London Hospital, E.

*Post.*

(137) Can you tell me of a cottage hospital or convalescent home where I could be taken as nurse? I have a slight training and am 38 years of age. Also, could you tell me how I could hear of vacancies in homes and schools for matrons?—*Anxious.*

You can only get what you want by advertisement. For nursing appointments the *Nursing Section* of THE HOSPITAL holds the first place; for schools, etc., the various scholastic and clerical papers are very good.

Will you kindly tell me how I can get a post as secretary to one of the London Hospitals? Is the post usually filled by a resident in the hospital, one of the sisters, for instance?—*G. MacR.*

The post of secretary is generally filled by a competent business man.

Will you kindly give me information how to obtain a post in a hospital in Paris?—*C. K.*

The only English hospital in Paris is the British Hertford Hospital, Rue de Villiers-Levallois Perret, Paris. Apply to the matron.

As my relatives have gone to live in Sweden, I should be glad if you will kindly tell me how to get an appointment in either a hospital or a home there. I have had two years' training in a large fever hospital?—*R. C. E.*

You will be much more likely to hear of a post if you join your relatives first, and seek work afterwards.

Can you suggest any form of home employment connected with nursing for a trained nurse who has to rest from active work until September?—*Crock.*

It is extremely difficult to find anything to do that will enable the nurse to rest. Perhaps some of our readers can suggest something, which presumably must be profitable.

*Sneezing.*

(138) Will you kindly tell me of anything that will stop a bad fit of sneezing?—*Windflower.*

An incidental fit of sneezing may sometimes be stopped by pinching the nose, but if sneezing becomes so frequent a trouble as to verge upon disease, consult a medical practitioner.

*Patent.*

(139) I wish to patent a cure for a disease, to whom should I apply for information?—*Willing.*

Apply the Patent Office, 25 Southampton Buildings, W.C., or talk it over with some wise friend, who will probably dissuade you from throwing your money away.

*District Nursing.*

(140) Will you kindly tell me to whom to apply for information respecting (1) the Queen Victoria's Jubilee Institute for Nurses, and (2) the Holt-Ockley Nursing Association?—*C. C. P. and W. D.*

Apply to the General Superintendent, St. Katherine's Precincts, Regent's Park, N.W., and (2) to the Secretary, the Affiliated Benefit Nursing Associations, 12 Buckingham Palace Road, S.W.

*Quarantine.*

(141) Will you kindly say what is the time specified by the Local Government Board for quarantine after nursing small-pox?—*W. M. B.*

Fifteen days is the usual quarantine.

*Steriliser.*

(142) Will the Editor kindly tell me where the best steriliser for towels and sponges may be obtained and the price. Only a small one for district use and occasional operations is required.—*A. A. H.*

Any of the firms of surgical appliances will be glad to supply you with particulars of sterilisers and then you can select the one which suits your purpose best.

*School.*

(143) Could you tell me if there is a school where a little boy of ten could be placed? His friends could pay 6s. weekly.—*District Nurse.*

You will find a list of schools in the "Englishwoman's Year-Book," price 2s. 6d.

*Norland Institute.*

(144) Will you kindly give me the address of the Norland Institute for training nurses for children?—*K. P.*

The address of the Norland Institute is 10 Pembridge Square, W.

*Lepers.*

(145) Will you kindly tell me to whom I should apply concerning nursing lepers in India or the Colonies? Would Government employ me, and are there any other agencies besides missionary societies?—*E. C. B.*

You will find a list of the leper hospitals in "Burdett's Hospitals and Charities." It will be extremely difficult to get work unless you are personally known to the local hospital authorities.

*Help.*

(146) Will you kindly inform me if there is any fund that would enable me to bring to justice a landlady who has maliciously told falsehoods to my patient's wife, which have caused me to be dismissed at a moment's notice?—*Grace.*

There is no private fund to help a nurse in taking proceedings for libel.

*Training School.*

(147) I should be grateful if you would kindly let me know whether the Wandsworth and Clapham Union Infirmary is a recognised training school for nurses.—*N. U.*

Certainly; see the *Nursing Section* of THE HOSPITAL, for July 26th, 1902.

*Abroad.*

(148) I want to go abroad to nurse for the winter. Will you be so kind as to give me addresses of nursing homes in Rome, San Remo, and Cannes?—*E. R.*

Rome, the Anglo-Nursing Home, 265 Via Nomentana; and San Remo, the Institute for Trained English Nurses, Sunny Bank.

**Standard Books of Reference.**

**"The Nursing Profession: How and Where to Train." 2s. net; post free 2s. 4d.**

**"Burdett's Official Nursing Directory." 3s. net; post free, 3s. 4d.**

**"Burdett's Hospitals and Charities." 5s.**

**"Hospital Expenditure: The Commissariat." 2s. 6d.**

"The Nurses' Dictionary of Medical Terms." Cloth, 2s.; leather, 2s. 6d. net.; post free, 2s. 8d.

**"Burdett's Series of Nursing Text-Books." 1s. each.**

**"A Handbook for Nurses." (Illustrated). 5s.**

**"The Physiological Feeding of Infants." 1s.**

**"The Physiological Nursery Chart." 1s.; post free, 1s. 3d.**

**All these are published by the SCIENTIFIC PRESS, Ltd., and may be obtained through any bookseller or direct from the publishers, 28 and 29 Southampton Street, London, W.C.**

### Travel Notes.

By Our Travelling Correspondent.

CIX.—ON THE MOORS.

Boat of Garten has a little station so close to the hotel, that it was our evening amusement to step on to the platform to watch the evening train through to Perth, thereby keeping our grip on civilisation. From Garten you can visit the Cairngorm Mountains—Loch Morlich, the forest of Rothiemurchess, and many other charming spots. The Loch of Garten is beautiful, but not so grim and grand as Morlich, where the surrounding mountains cast indigo reflections in its depths. Loch-an-Eilan is only three miles from Aviemore station, and the walk thence enchanting. It is reported as being one of the last resorts of the vanishing osprey. On a little island are the remains of a castle, once the stronghold of the "Wolf of Badenoch," the fierce son of Robert II. Near here is a great opening into the mountains leading to Braemar, called the Larigghru Pass, which means "the forbidden way."

The Field of Culloden.

To get on to the marvellous Caledonian Canal, which splits the country from east to west, it is necessary to go to Inverness, where there is nothing to detain you. You must pass a night there, however, to catch the boat at 7.30 a.m., therefore reach Inverness the previous night in time to see Culloden. Go to the Muirtown Hotel, it is on the canal quay, and is convenient for an early start, and the owners are most kind and obliging and their charges moderate.

The last station before you reach Inverness is Culloden Moor, which is about half a mile from the battlefield, and you had better alight there, going on later to Inverness itself. If you drive to the battlefield from the city it is six miles, and I think the charge for a dog-cart was 12s. On the 16th of April, 1746, the grandson of James II. made his ill-advised attempt to turn the fortune of war in his favour, and we all know what that attempt cost him and his.

A sort of moral fog seems always to have obscured the mental faculties of that fascinating but ill-fated family. Why, for instance, should Prince Charlie have chosen to give battle on such a place as Drummossie Moor, as it is locally called—a place absolutely unsuited for such soldiers as the Highlanders, who fight best where broken ground is no bar to their trained agility, but giving every facility for their opponents (well mounted) to pursue and cut them down in the event of defeat.

A mile distant is Culloden House, where the unfortunate Prince slept on the eve of the battle, and up to 1897 this was full of Jacobite relics, but then, alas, on the death of Mr. Forbes, these all came to the hammer.

At one corner of the fatal field is a huge flat stone, which tradition says was the breakfast table of the Duke of Cumberland. It is very likely, for in all probability the cruel but able commander of the Hanoverian forces was early on the scene, perhaps slept there all night, whilst the fated Prince, too sanguine and easy going, slept at Culloden House, and dreamt that the Divine right of kings would make all well on the morrow.

It has always been said that one of the determining causes of his defeat was the marching away in dudgeon of one of the largest Highland clans because they were not allowed to occupy what was considered the greatest point of danger.

In the centre of the field is a huge cairn, raised to commemorate the battle, and there are rough stones inscribed with the names of the different clans who perished, almost to a man, in the encounter; these lie at the head of the long trenches in which the self-willed, mutinous, quarrelsome, and vain, but always gallant and devoted, Highlanders sleep their last sleep. There is something very touching and impressive in this lonely field, which is one vast graveyard, simply marked by its cairn and the few granite slabs, with no inscription and no statement, simply the names of the doomed clans who followed the fortunes of their hereditary prince.

Inverness, at present a dull modern town, must in those days have witnessed some stirring scenes; probably many fugitives were hidden in its lanes and wynds, hopeful of escaping the troopers of the Duke of Cumberland.

The Caledonian Canal.

I fear I must not linger to say much of this delightful journey. It will take you the entire day to reach Ballachulish, which I propose should be your next point. Progress is slow on account of the many locks to be negotiated, but I am sure you will not find the time long. At Fort Augustus there is a modern but very picturesque Benedictine monastery, built on the site of the old fort, which in 1746 served as a prison for a large number of the followers of the Prince, among others Lord Lovat, who was removed thence and executed on Tower Hill with Balmerino and Kilmarnock.

North Ballachulish.

This place you will reach in the evening, landing at Onich. Though I have been a great traveller in a humble way I have always classed Ballachulish in my mind as one of the most beautiful spots on earth; the Morven range of mountains—blue, red, or yellow, according to weather, to the right; the Pass of Glencoe to the left, the mountain of Creag-Gorm (the blue hill) in front, and at one's feet the "narrows," across which the ferry is for ever plying. There is only one hotel in North Ballachulish, but it is an ideal one, where you feel as if with friends. From it you descend a sharply-pitched little causeway to the ferry and here many funny and picturesque scenes are witnessed; what I liked best was to watch sheep being brought over in charge of the shepherd and his trusty and clever dog. Ballachulish is full of local and historic interest, something of which I hope to tell you next week.

### TRAVEL NOTES AND QUERIES.

Rules in Regard to Correspondence for this Section.—All questioners must use a pseudonym for publication, but the communication must also bear the writer's own name and address as well, which will be regarded as confidential. All such communications to be addressed "Travel Editor, 'Nursing Section of *The Hospital*,' 28 Southampton Street, Strand." No charge will be made for inserting and answering questions in the inquiry column, and all will be answered in rotation as space permits. If an answer by letter is required, a stamped and addressed envelope must be enclosed, together with 2s. 6d., which fee will be devoted to the objects of "The Hospital" Convalescent Fund. Any inquiries reaching the office after Monday cannot be answered in "The Hospital" of the current week.

Addresses in Hanover and France (J. H.).—Thanks for your valuable information; it is sure to be most useful to those you kindly wish to benefit. I have filed your letter.

South Brittany for 15 Days (Nemo).—I am glad my former instructions were found useful, and pleased to hear from you. A fortnight is rather short for South Brittany, it is such slow travelling, a night to go over, and it is problematic whether you catch a train that will take you south in one day, say to Vannes or Auray. I think I should start from Vannes, because it is architecturally more interesting than Auray which, together with Quiberon, can be visited from Vannes. It is a question whether to go next to Quimper or Quimperlé, both are delightful, and both good centres for excursions. Quimper is the capital of Finisterre, and is the more important place; they are about 30 miles apart, and can be visited from one another. Visit also Douarnenez from Quimper, then strike north-east to Pontivy, and home *viâ* St. Brieuc and St. Malo.

